# Characterization of elite controllers with an undetectable intact HIV DNA reservoir

**DOI:** 10.1172/JCI202577

**Published:** 2026-02-17

**Authors:** Jana Blazkova, Brooke D. Kennedy, Jesse S. Justement, Victoria Shi, Adeline Sewack, Maegan R. Manning, Sonali Dasari, Kathleen Gittens, Susan Moir, Mark Connors, Stephen A. Migueles, Tae-Wook Chun

**Affiliations:** 1 Laboratory of Immunoregulation, National Institute of Allergy and Infectious Diseases (NIAID) and; 2 Clinical Center, NIH, Bethesda, Maryland, USA.

**Keywords:** AIDS/HIV, Immunology, Virology, Cellular immune response, Molecular genetics, T cells

## Abstract

Some people with HIV control the virus without medication. This study identified a rare group lacking detectable HIV reservoirs, offering important clues for developing future cures.

**To the Editor:** Elite controllers (ECs) are people with HIV (PWH) who can naturally control HIV infection without antiretroviral therapy (ART). A better understanding of the mechanisms for this control could inform novel therapeutic strategies for achieving a cure for HIV. Multiple factors, including the HIV DNA reservoir size, the landscape of viral integration, viral fitness, protective HLA alleles, and potent T cell responses, may contribute to elite control (1, 2). Previous studies have demonstrated significantly smaller HIV DNA reservoirs, including intact proviral DNA (IPD), in ECs compared with PWH undergoing chronic ART (3, 4). In this study, we examined 34 ECs to identify and characterize those without detectable IPD and to elucidate the mechanisms underlying their exceptional virologic control.

We first examined virological parameters, including intact, defective, total HIV DNA, and cell-associated HIV RNA. Surprisingly, 13 ECs had undetectable IPD (EC-IPD0), whereas 21 ECs had detectable IPD (EC-IPD^+^) (Figure 1A). When the ECs were stratified by IPD levels, there were no significant differences in age, sex, duration of infection after diagnosis, or CD4^+^ and CD8^+^ T cell counts between the groups (Supplemental Table 1; supplemental material available online with this article; https://doi.org/10.1172/JCI202577DS1). However, the total (*P* < 0.0001) and 3′ defective (*P* = 0.0319) HIV DNA levels were significantly lower in the EC-IPD0 group compared with the EC-IPD^+^ group, while levels of 5′ defective HIV DNA (*P* = 0.0834) and cell-associated HIV RNA (*P* = 0.0770) did not differ between the groups (Figure 1A). The frequencies of cells harboring replication-competent HIV were significantly lower in the EC-IPD0 compared with the EC-IPD^+^ participants (*P* = 0.0173; Figure 1B), indicating that EC-IPD0 participants maintained extraordinarily low numbers of productively infected cells. Notably, the HLA-B*57 haplotype was significantly more prevalent in the EC-IPD0 group (61.5%) than in the EC-IPD^+^ group (33.3%) (*P* = 0.0324; Figure 1C and Supplemental Table 1). Of note, confirmatory HIV antibody tests yielded indeterminate results in 3 ECs from the EC-IPD0 group, whereas all participants in the EC-IPD^+^ group tested positive (Supplemental Table 2).

To gain further insights into the low infectious HIV burden of the EC-IPD0 participants, we conducted high-dimensional spectral flow cytometry to examine T cell immunophenotypes. On the basis of a panel of 28 cell-surface markers, we identified significantly reduced frequencies of effector memory (Tem) (*P* = 0.0397) and effector memory RA (Temra) (*P* = 0.0040) CD4^+^ T cells, as well as significantly elevated frequencies of central memory CD8^+^ T cells (Tcm) (*P* = 0.0154) in the EC-IPD0 compared with the EC-IPD^+^ group (Figure 1D). Additional analysis using the FlowSOM algorithm identified 2 of the 30 meta-clusters that were significantly reduced in the EC-IPD0 group (Figure 1E). These included Tem/terminally differentiated (Tem/Ttd) CD8^+^ T cells expressing senescent (CD57) and exhaustion (2B4) markers (cluster 12, *P* = 0.0406) and transitional/Tem (Ttm/Tem) cytotoxic (Tc1) CD8^+^ T cells expressing high levels of the activation markers CD38 and HLA-DR (cluster 25, *P* = 0.0242). Antigen-specific T cell analysis showed comparable levels of polyfunctional HIV Gag-specific CD4^+^ (*P* = 0.5937) and CD8^+^ T cells (*P* = 0.2800) between the groups (Figure 1F). Finally, we measured the levels of the biomarkers PD-L1, CXCL10, IL-2Ra, IL-6, granzyme B, perforin, and RANTES in the plasma of the study participants. Among them, only IL-6 levels were significantly elevated in EC-IPD0 versus EC-IPD^+^ participants (*P* = 0.0154; Supplemental Figure 1). Of note, analyses of innate immune cells, including NK and plasmacytoid DCs, revealed no differences between the groups (Supplemental Figure 2).

The identification of a sizable number of ECs with undetectable IPD could potentially advance our understanding of the mechanisms of HIV control without ART. They exhibit extraordinarily low levels of intact and infectious HIV, suggesting the near-complete elimination of infected cells. Our findings indicate several distinguishing immunologic features in EC-IPD0 participants, including enrichment of the HLA-B*57 haplotype, reduced frequencies of effector CD4^+^ and exhausted/activated CD8^+^ T cells. In particular, the predominance of the HLA-B*57 haplotype in EC-IPD0 aligns with previous findings demonstrating its association with superior HIV control, driven by a strong CD8^+^ T cell response and subsequent clearance of infected cells (2, 5). However, the functionality of CD8^+^ T cells (i.e., proliferative and cytotoxic responses to additional HIV antigens) between the 2 groups requires further investigation. Our data further suggest that this genetic trait within the EC cohort may facilitate enhanced elimination of the persistent HIV reservoir, in addition to effective control of plasma viremia in the absence of ART. Additionally, the reduced frequency of terminally differentiated CD8^+^ T cells expressing exhaustion markers suggests the preservation of functional immune responses, potentially enabling the elimination of detectable HIV reservoirs; however, this could be due to lower antigen exposure in the EC-IPD0 versus the EC-IPD^+^ group. The elevated IL-6 level in EC-IPD0 is intriguing (6), and its role in the clearance of infected cells warrants further investigation.

The limitations of our work include the small sample size, as well as the lack of longitudinal time points and lymphoid tissue analyses. Nonetheless, we demonstrated that some ECs achieved near-complete elimination of infectious HIV reservoirs and identified correlates of this remarkable virologic control. Our findings provide important insights into the mechanisms underlying the elimination of persistent HIV reservoirs in the EC population that can apply to the development of therapeutic strategies aimed at achieving an HIV cure.

## Conflict of interest

The authors have declared that no conflict of interest exists.

## Funding support

This work is the result of NIH funding and is subject to the NIH Public Access Policy. Through acceptance of this federal funding, the NIH has been given a right to make the work publicly available in PubMed Central.

- This research was supported by the Intramural Research Program of the National Institute of Allergy and Infectious Diseases (NIAID), NIH. The contributions of the NIH authors are considered Works of the United States Government. The findings and conclusions presented in this paper are those of the authors and do not necessarily reflect the views of the NIH or the U.S. Department of Health and Human Services.

## Supplementary Material

Supplemental data

Supporting data values

## Figures and Tables

**Figure 1:**
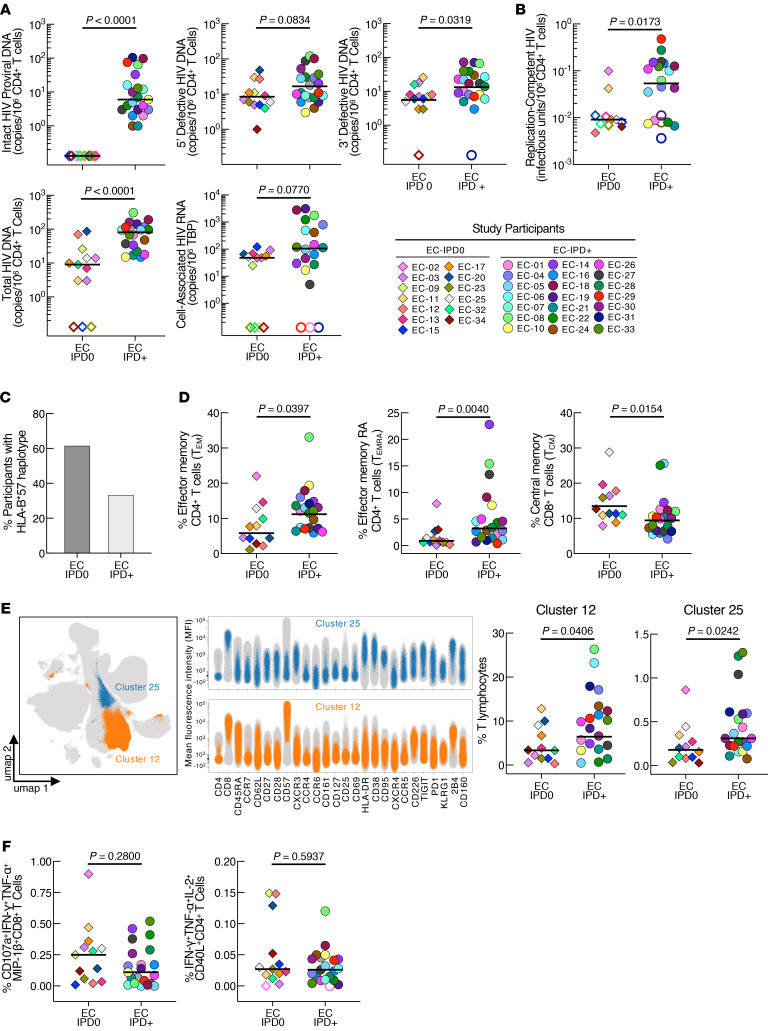
HIV reservoirs and immune parameters in ECs with undetectable (EC-IPD0) versus detectable (EC-IPD^+^) intact HIV DNA. Comparisons of (**A**) intact, defective, and total HIV DNA, cell-associated HIV RNA, and (**B**) replication-competent virus. (**C**) Proportion of participants with HLA-B*57 phenotype. (**D**) Frequencies of Tem and Temra CD4^+^ T cells and Tcm CD8^+^ T cells. (**E**) Uniform manifold approximation and projection (UMAP) of T cell clusters identified by FlowSOM clustering, MFI plots showing the level of expression of individual markers in 2 significantly different clusters, cluster 12 and cluster 25, and comparison of the frequencies of those clusters in T lymphocytes. (**F**) Comparisons of frequencies of HIV Gag-specific polyfunctional CD4^+^ and CD8^+^ T cells. Open symbols indicate values under the limit of detection. Black bars represent the median values. *P* values were determined using the Mann-Whitney *U* test.

## Version 1 02/17/2026

In-Press Preview

## Version 2 04/15/2026

Electronic publication
