# Supplementary material for: Characterization of elite controllers with an undetectable intact HIV DNA reservoir
Source: J Clin Invest. 2026 Feb 17;136(8):e202577. doi: 10.1172/JCI202577 (PMC13078864; doi:10.1172/JCI202577)
Supplement: Supplemental data [file jci-136-202577-s032.pdf]

Supplemental Materials

Supplemental Methods

Sex as a biological variable

Our study examined male and female participants, and similar findings are reported for both sexes.

Study participants

We studied 34 elite controllers (ECs): 13 with undetectable (EC-IPD0) and 21 with detectable intact proviral DNA (EC-IPD+; Supplemental Table 1). All study participants were ART-naïve, had plasma viremia measurements below the limits of detection, maintained $CD4^+$ T-cell counts >450 cells/mm^3, and had no AIDS-defining illnesses at the time of study. The median duration of HIV infection was 17.8 years (range 4.4-30.8) and 23.5 years (range 4.0-34.3) for the EC-IPD0 and EC-IPD+, respectively (*P*=0.2722, Supplemental Table 1).

Quantitation of HIV reservoirs

The frequency of $CD4^+$ T cells carrying intact and defective HIV DNA was measured by modified intact proviral DNA assay (IPDA) (1, 2) using digital PCR (QIAGEN). The intact HIV proviral DNA measured by this assay may include defective HIV DNA that does not contribute to the infectious viral reservoir (3). Genomic DNA was isolated with the QIAamp DNA Mini Kit (QIAGEN) from purified $CD4^+$ T cells and amplified with HIV-specific and *RPP30*-specific primers and probes. For HIV *gag*, the following primers and probes were used (2): (5'-GACTAGCGGAGGCTAGAAGGAGAGA-3' (5' primer), 5'-CTAATTCTCCCCCGCTTAATAYTGACG-3' (3' primer), 5'-6FAM-

A+T+GGG+TG+CGAGA-IABkFQ-3' (LNA probe) For HIV *env*, the following primers and probes were used: 5'- AGTGGTGCAGAGAGAAAAAAGAGC-3' (5' primer), 5'-GTCTGGCCTGTACCGTCAGC-3' (3' primer), 5'-VIC- CCTTGGGTTCTTGGGA-MGB-3' (probe) and 5'- CCTTAGGTTCTTAGGAGC-MGB-3' (unlabeled hypermutated probe). For housekeeping gene *RPP30*, the following primers and probes were used: 5'-GATTTGGACCTGCGAGCG-3' (RPP30-1 5' primer), 5'- GCGGCTGTCTCCACAAGT-3' (RPP30-1 3' primer), 5'-6FAM-TTCTGACCTGAAGGCTCTGCGC-IABkFQ-3' (RPP30-1 probe), 5'- GTGTGAGTCAATCACTAGACAGAA-3' (RPP30-2 5' primer), 5'-AAACTGCAACAACATCATAGAGC-3' (RPP30-2 3' primer), and 5'-HEX-AGAGAGCAACTTCTTCAAGGGCCC-IABkFQ-3' (RPP30-2 probe). The HIV-specific reactions were performed in 4-16 technical replicates, and the RPP30-specific reactions were performed in 4 technical replicates. Copy numbers were normalized per $1x10^6$ $CD4^+$ T cells, and intact HIV DNA copies were adjusted with the DNA shearing index (DSI) based on a ratio of double-positive RPP30 partitions. The positive and negative controls for the IPDA included genomic DNA from PBMCs and $CD4^+$ T cells from HIV-negative donors, spiked with or without serially diluted genomic DNA from the ACH-2 cell line. The limit of detection was 1-2 copies per 10^6 cells.

The frequency of $CD4^+$ T cells carrying HIV DNA was measured by digital PCR (QIAGEN) and primers and probe specific for the 5' long terminal repeat (LTR) region. Genomic DNA was isolated from purified $CD4^+$ T cells (QIAGEN) and digested (MscI; New England BioLabs) prior to PCR amplification with HIV-specific and RPP30-specific primers and probes. For amplification of the HIV LTR, the following primers and probes were used: 5'-GRAACCCACTGCTTAAGCCTCAA -3' (5' primer) and 5'-

TGTTCGGGCGCCACTGCTAGAGA -3' (3' primer), and 5'-6FAM-AGTAGTGTGTGCCCGTCTGTT-IABkFQ-3' (probe). For amplification of *RPP30*, the following primers and probes were used: 5'-GATTTGGACCTGCGAGCG-3' (5' primer), 5'-GCGGCTGTCTCCACAAGT-3' (3' primer), and 5'-HEX-TTCTGACCTGAAGGCTCTGCGC-IABkFQ-3' (probe). HIV DNA copy numbers were normalized per $1x10^6$ $CD4^+$ T cells.

The level of cell-associated HIV RNA was measured by RT-PCR and digital PCR (QIAGEN). Total RNA was isolated from purified $CD4^+$ T cells using the RNeasy Mini Kit (QIAGEN) according to the manufacturer's instructions. Complementary DNA (cDNA) was synthesized with qScript XLT cDNA Master Mix (Quanta Biosciences) according to the manufacturer's instructions. cDNA products were quantified using HIV-specific and TATA box-binding protein (TBP)-specific primers and probes. For HIV RNA (unspliced) (4), the following primers and probes were used: 5'- TCTCTAGCAGTGGCGCCCGAACA-3' (5' primer), 5'-TCTCCTTCTAGCCTCCGCTAGTC-3' (3' primer), 5'-6FAM-CAAGCCGAGTCCTGCGTCGAGAG-IABkFQ-3' (probe). For *TBP*, the following primers and probes were used: 5′-CACGAACCACGGCACTGATT-3′ (5' primer), 5′-TTTTCTTGCTGCCAGTCTGGAC-3′ (3' primer), and 5′-HEX-TGTGCACAGGAGCCAAGAGTGAAGA-IABkFQ-3′ (probe). Copy numbers of HIV RNA were normalized per $1x10^6$ copies of the housekeeping gene *TBP*.

Levels of $CD4^+$ T cells carrying replication-competent HIV were determined by a quantitative co-culture assay using serially diluted (1,000,000, 200,000, 40,000, 8,000, 1,600, and 320 cells per well) and replicates of $5x10^6$ $CD4^+$ T cells from each study participant. Each well was incubated with anti-CD3 antibodies and irradiated PBMCs from healthy HIV-negative donors. After 1 day of incubation, $1x10^6$ CD8-depleted and anti-CD3-stimulated PBMCs from

HIV-negative donors were added to each well, followed by removal of cell suspensions and replenishment with fresh medium containing IL-2 every 3 days. HIV p24 ELISA was performed on the culture supernatants to identify wells containing replication-competent HIV.

Immune phenotyping by flow cytometry

Cryopreserved PBMCs were thawed, washed, and stained with the viability reagent Zombie NIR (Biolegend #423106), fluorophore-conjugated antibodies, and Brilliant Stain Buffer Plus (BD Biosciences #566385). Antibodies used for T-cell phenotyping included: CD3-BUV805 (clone SK7, BD Biosciences #612893), CD4-BUV395 (clone RPA-T4, BD Biosciences #564724), CD8-BUV737 (clone SK1, BD Biosciences #612754), CD45RA-PerCP-Cy5.5 (clone HI100, BD Biosciences #563429), CCR7-V450 (clone 150503, BD Biosciences #560863), CXCR4-BV510 (clone 12G5, Biolegend #306536), CCR4-BUV615 (clone 1G1, BD Biosciences #613000), CCR5-BV650 (clone 3A9, BD Biosciences #564999), CXCR3-BV711 (clone G025H7, Biolegend #353732), CCR6-APC/Fire750 (clone G034E3, Biolegend #353443), CD27-BV421 (clone O323, Biolegend #302824), CD28-BV480 (clone CD28.2, BD Biosciences #566110), CD127-BUV563 (clone eBioRDR5, eBioscience #365-1278-42), CD38-APC/Fire810 (clone HB-7, Biolegend #356644), CD226-BUV496 (clone DX11, BD Biosciences #749935), CD69-BV605 (clone FN50, Biolegend #310938), CD25-BV785 (clone BC96, Biolegend #302638), CD95-PerCP-eFluor 710 (clone DX2, eBioscience #46-0959-42), KLRG1-APC (clone 2F1/KLRG1, Biolegend #138412), PD-1-PE-Cy7 (clone EH12.1, BD Biosciences #561272), CD160-Alexa Fluor 647 (clone BY55, BD Biosciences #562362), CD161-PE-Cy5 (clone HP-3G10, Biolegend #339951), CD62L-R718 (clone DREG-56, BD Biosciences #567988), HLA-DR-Super Bright 436 (clone LN3, eBioscience #62-9956-42), TIGIT-PE-eFluor 610 (clone MBSA43, eBioscience #61-9500-42), 2B4-PE (clone C1.7, eBioscience #12-5838-

42), CD45-SparkBlue550 (clone 2D1, Biolegend #368550), and CD57-BB515 (clone NK-1, BD Biosciences #565285). Data were collected on a spectral flow cytometer Cytek Aurora using the SpectroFlo Software (Cytek Biosciences) and analyzed using FlowJo version 10.10.0 and the OMIQ platform (omiq.ai).

High-dimensional analysis of flow cytometry data

Uniform Manifold Approximation and Projection (UMAP) dimensionality reduction and FlowSOM clustering algorithms were used via the OMIQ platform (omiq.ai) to analyze the flow cytometry data. An equal sampling of 150,000 CD3^{+} T cells from each FCS data file was used for the analysis. The following markers: CD4, CD8, CD45RA, CCR7, CD62L, CD27, CD28, CD57, CXCR3, CCR4, CCR6, CD161, CD127, CD25, CD69, HLA-DR, CD38, CD95, CXCR4, CCR5, CD226, TIGIT, PD-1, KLRG1, 2B4 and CD160 were used for the UMAP embedding and FlowSOM clustering with the number of meta-clusters set to 30.

Intracellular Cytokine Staining (ICS) assay

The frequencies of HIV Gag-specific CD4^{+} and CD8^{+} T cells were assessed by intracellular cytokine staining. Cryopreserved PBMCs were thawed, rested overnight, and incubated for 6 hours at 37°C with overlapping HIV-1 Gag peptides (NIH AIDS Reagent Program), Brefeldin A (Sigma-Aldrich), and anti-CD107a-BV510 antibody. Cells were stained with Zombie NIR (Biolegend #423106), and the following fluorophore-conjugated antibodies for surface markers: CD3-BUV805 (clone SK7, BD Biosciences #612893), CD4-BUV395 (clone RPA-T4, BD Biosciences #564724), and CD8-BUV737 (clone SK1, BD Biosciences #612754). Stained cells were fixed with 1x Lysing Solution (BD Biosciences) and permeabilized with 1x Permeabilization Solution 2 (BD Biosciences) prior to incubation with the following intracellular

antibodies: IFN-γ-APC (clone B27, BD Biosciences #554702), TNF-α-BV650 (clone MAb11, BD Biosciences #563418), MIP-1ß-PE (clone D21-1351, BD Biosciences #550078), IL-2-PerCP-eFluor710 (clone MQ1-17H12, eBioscience #46-7029-42), and CD40L-BV421 (clone TRAP1, BD Biosciences #563886). Data were acquired using the SpectroFlo software (Cytek Biosciences) on the Cytek Aurora cytometer, and analysis was performed using FlowJo version 10.10.0.

Examination of Biomarkers in plasma

Levels of plasma-associated biomarkers were determined using the ELLA (ProteinSimple) platform as instructed by the manufacturer.

Detection of antibodies against HIV-1/2 in plasma

Antibodies against HIV-1/2 in plasma were detected using the Geenius HIV 1/2 Supplemental Assay (Bio-Rad) according to the manufacturer's instructions using plasma collected at the time of study.

Statistical analysis

P values for comparison of the 2 groups were determined by the Mann-Whitney test. *P* values for comparison of demographic parameters (sex and race) and HLA types were calculated using Fisher's exact test. All tests were performed with Prism 10.4.1 (GraphPad).

Study approval

Blood products were obtained in accordance with the protocol approved by the Institutional Review Board of the NIAID, NIH. All participants provided written informed consent.

Data availability

Data are available in the “Supporting data values” XLS file or from the corresponding author upon request.

Supplemental Table 1. Clinical characteristics of study participants.

	**EC-IPD0** (n=13)	**EC-IPD+** (n=21)	***P*-value**[a]
**Sex**, number (%)			
Male	8 (61.5)	14 (66.7)	>0.9999[b]
Female	5 (38.5)	7 (33.3)	
**Age** (years)			
Median (interquartile range)	54 (50.5-61.5)	55 (45-63.5)	0.6426
Range	41-70	32-78	
**Race**, number (%)			
African American	5 (38.5)	7 (33.3)	0.8656[b]
Caucasian	7 (53.8)	13 (61.9)	
Hispanic	1 (7.7)	1 (4.8)	
**$CD4^+$ T cell count** (cells/mm^3)			
Median (interquartile range)	896 (691-1,321)	778 (607-1,056)	0.3087
Range	467-1,728	491-1,891	
**$CD8^+$ T cell count** (cells/mm^3)			
Median (interquartile range)	613 (360-826)	588 (380-824)	0.9443
Range	303-1,116	281-1,400	
**Duration of infection following diagnosis** (years)			
Median (interquartile range)	17.8 (7.9-24.2)	23.5 (8.8-31.7)	0.2722
Range	4.4-30.8	4.0-34.3	
**HLA Types**, number (%)			
HLA B27	1 (7.7)	4 (19.1)	0.6272[b]
HLA B57	8 (61.5)	7 (33.3)	**0.0324**[b]

a Mann-Whitney test

b Fisher's exact test

Supplemental Table 2. Assessment of antibodies against HIV-1.

Group	Participant ID	HIV-1				Interpretation
		p31	gp160	p24	gp41	
EC-IPD0	EC-02	+	+	+	+	Positive
	EC-03	+	+	+	+	Positive
	EC-09	-	+	-	+	Positive
	EC-11	+	+	+	+	Positive
	EC-12	-	-	-	+	**Indeterminate**
	EC-13	-	+	+	+	Positive
	EC-15	-	-	-	+	**Indeterminate**
	EC-17	+	+	+	+	Positive
	EC-20	+	+	+	+	Positive
	EC-23	-	+	-	+	Positive
	EC-25	-	+	+	+	Positive
	EC-32	+	+	+	+	Positive
	EC-34	-	-	-	+	**Indeterminate**
EC-IPD+	EC-01	-	+	+	+	Positive
	EC-04	+	+	+	+	Positive
	EC-05	+	+	+	+	Positive
	EC-06	+	+	+	+	Positive
	EC-07	-	+	-	+	Positive
	EC-08	-	+	-	+	Positive
	EC-10	-	+	+	+	Positive
	EC-14	+	+	+	+	Positive
	EC-16	+	+	+	+	Positive
	EC-18	-	+	+	+	Positive
	EC-19	+	+	+	+	Positive
	EC-21	-	+	+	+	Positive
	EC-22	+	+	+	+	Positive
	EC-24	-	+	+	+	Positive
	EC-26	-	+	+	+	Positive
	EC-27	+	+	+	+	Positive
	EC-28	+	+	+	+	Positive
	EC-29	-	+	+	+	Positive
	EC-30	+	+	+	+	Positive
	EC-31	+	+	+	+	Positive
	EC-33	+	+	+	+	Positive

Supplemental Figure 1. Biomarkers in the plasma of elite controllers (EC) with undetectable (EC-IPD0) versus detectable (EC-IPD+) intact HIV DNA.

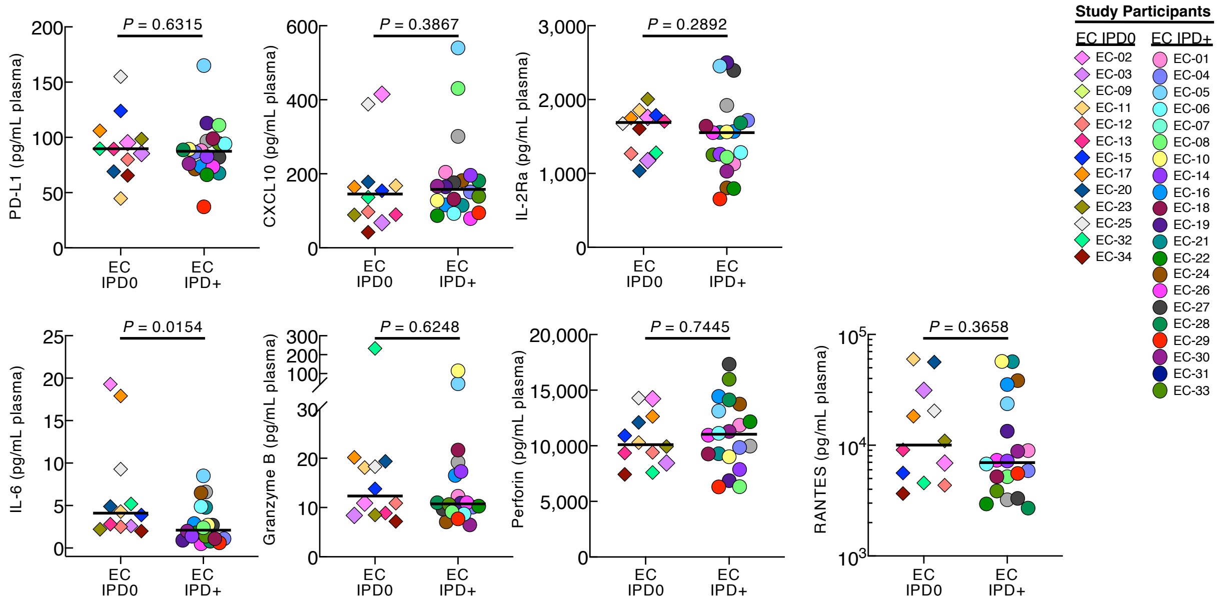

Comparison of PD-L1, CXCL10, IL-2Ra, IL-6, granzyme B, perforin, and RANTES levels in the plasma of study participants. Black bars represent the median values. *P* values were determined using the Mann-Whitney test.

Supplemental Figure 2. Innate immune characteristics in elite controllers (EC) with undetectable (EC-IPD0) versus detectable (EC-IPD+) intact HIV DNA.

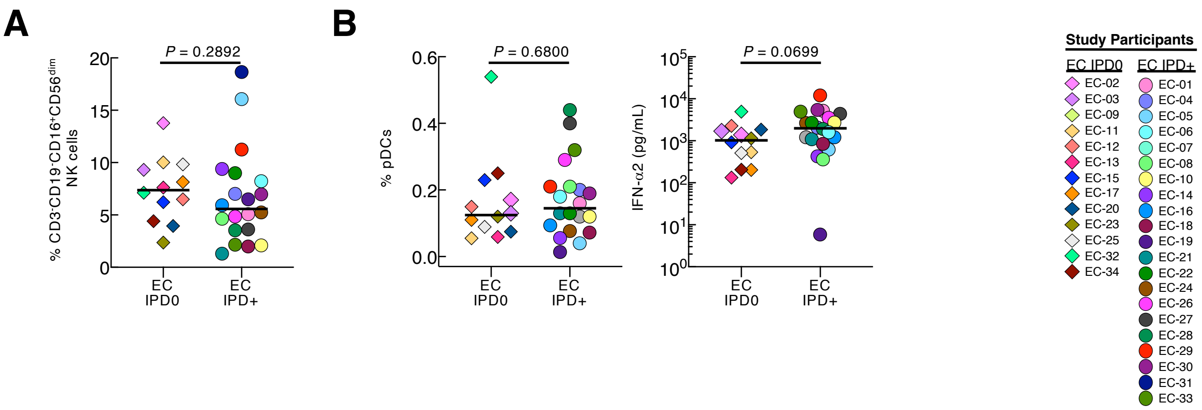

(**A**) Frequencies of CD3^{-}CD19^{-}CD16^{+}CD56^{dim} natural killer (NK) cells. (**B**) Frequencies of plasmacytoid dendritic cells (pDC; left) and levels of IFNα-2 expression upon CpG stimulation of PBMCs per mL of cell-culture supernatant (right). Black bars represent the median values. *P* values were determined using the Mann-Whitney test.

Acknowledgements

We thank the volunteers for their participation in this study.

Author contributions

JB, BDK, and TWC designed the research. JB, BDK, JSJ, VS, AS, MRM, and TWC performed the research. SD, KG, MC, and SAM contributed research materials. JB, BDK, SM and TWC analyzed the data. JB and TWC wrote the manuscript. All authors reviewed and approved the manuscript.

Supplemental references

1. Bruner KM, Wang Z, Simonetti FR, Bender AM, Kwon KJ, Sengupta S, et al. A quantitative approach for measuring the reservoir of latent HIV-1 proviruses. *Nature.* 2019;566(7742):120-5.
2. Levy CN, Hughes SM, Roychoudhury P, Reeves DB, Amstuz C, Zhu H, et al. A highly multiplexed droplet digital PCR assay to measure the intact HIV-1 proviral reservoir. *Cell Rep Med.* 2021;2(4):100243.
3. Kennedy BD, Blazkova J, Justement JS, Shi V, Rai MA, Manning MR, et al. Comprehensive analysis of HIV reservoirs in elite controllers. *J Clin Invest.* 2023;133(3).
4. Saksela K, Muchmore E, Girard M, Fultz P, and Baltimore D. High viral load in lymph nodes and latent human immunodeficiency virus (HIV) in peripheral blood cells of HIV-1-infected chimpanzees. *J Virol.* 1993;67(12):7423-7.
